# Biomechanical comparative finite element analysis between a conventional proximal interphalangeal joint flexible hinge implant and a novel implant design using a rolling contact joint mechanism

**DOI:** 10.1186/s13018-023-04477-y

**Published:** 2023-12-19

**Authors:** Yong-Jae Kim, Hyun-ah Bae, Seok Woo Hong

**Affiliations:** 1 School of Electrical, Electronics & Communication Engineering, Korea University of Technology and Education, 1600, ChungJeol-Ro, Dongnam-Gu, Cheonan, 31253 Republic of Korea; 2 Department of Orthopaedic Surgery, Kangbuk Samsung Hospital, Sungkyunkwan University School of Medicine, 29, Saemunan-Ro, Jongno-Gu, Seoul, 03181 Republic of Korea

**Keywords:** Proximal interphalangeal joint prosthesis, Flexible hinge implant, Rolling contact joint mechanism, Finite element analysis, Von-Mises stress, Von-Mises strain, Moment reactions

## Abstract

**Background:**

The rolling contact joint (RCJ) mechanism is a system of constraint that allows two circular bodies connected with flexible straps to roll relative to one another without slipping. This study aims to compare the biomechanical characteristics between the conventional proximal interphalangeal joint (PIPJ) flexible hinge (FH) implant and the novel PIPJ implant adopting a RCJ mechanism during PIPJ range of motion using finite element (FE) analysis.

**Methods:**

The three-dimensional (3D) surface shape of a conventional PIPJ FH implant was obtained using a 3D laser surface scanning system. The configuration and parameters of the novel PIPJ implant were adapted from a previous study. The two implants were assumed to have the same material characteristics and each implant was composed of a hyperelastic material, silicone elastomers. The configuration data for both implants were imported to a computer-aided design program to generate 3D geometrical surface and hyperelastic models of both implants. The hyperelastic models of both implants were imported into a structural engineering software to produce the FE mesh and to perform FE analysis. The FE analysis modeled the changes of mechanics during flexion–extension motion between 0° and 90° of two PIPJ implants. The mean and maximum values of von-Mises stress and strain as well as the total moment reaction based on the range of motion of the PIPJs were calculated. The mean values within the PIPJ’s functional range of motion of the mean and maxinum von-Mises stress and strain and the total moment reaction were also determined.

**Results:**

The maximum values for the von-Mises stress, and strain, as well as the total moment reactions of the conventional PIPJ FH and novel PIPJ implants were all at 90° of PIPJ flexion. The maximum value of each biomechanical property for the novel PIPJ implant was considerably lower compared with that of the conventional PIPJ FH implant. The mean values within the PIPJ’s functional range of motion of the maximum von-Mises stress and strain for the novel PIPJ implant was approximately 6.43- and 6.46-fold lower compared with that of the conventional PIPJ FH implant, respectively. The mean value within a PIPJ’s functional range of motion of the total moment reaction of the novel PIPJ implant was approximately 49.6-fold lower compared with that of the conventional PIPJ FH implant.

**Conclusions:**

The novel PIPJ implant with an RCJ mechanism may offer improved biomechanical performance compared with conventional PIPJ FH implant.

**Supplementary Information:**

The online version contains supplementary material available at 10.1186/s13018-023-04477-y.

## Introduction

Following the introduction of the Swanson^®^ finger joint implant (Wright Medical Technology, Inc., Arlington, TN) in the 1960s, various flexible hinge (FH) implants have been developed [1]. Newly introduced typical FH implants include the NeuFlex^®^ silicone implant (Depuy, Warwaw, IN) and the Avanta® silicone implant (Avanta Orthopaedice, San Diego, CA) [2, 3]. Materials used in these implant are continuously improving, and the designs have gradually been modified to mimic human body biomechanics [4, 5]. Although FH implants are a lineage of hand implants, their biomechanics have not significantly changed from those of the original Swanson silicone implant [1]. In addition, the FH implants have a high probability of implant failure within a decade of implantation. [6, 7] Because proximal interphalangeal joint (PIPJ) arthroplasty using FH implant is reported to be performed in individuals around the age of 60 [8, 9], considering the average human lifespan, a minimum of two revision surgeries may be required after implantation. Furthermore, improvement in the range of motion is not significant compared with that before the operation [7, 10]. Thus, there is a need for new implants that offers greater longevity and considerably improved range of motion.

The rolling contact joint (RCJ) mechanism is a system of constraint that allows two circular bodies connected with flexible straps to roll relative to one another at the contact surface without slipping [11]. The authors have designed a novel PIPJ implant that mimics the human anatomy and biomechanical properties of PIPJ based on this mechanism [12]. This novel implant offers a greater range of motion compared with FH implants and provides sufficient stability via straps [13]. Furthermore, the novel RCJ implant allows flexion with minimal moment and has a wide cross-sectional area that can withstand relatively high compressive forces [12, 14]. Therefore, the PIPJ implant using the RCJ mechanism may be a good replacement for FH implants.

All FH implants, including the Swanson^®^ finger joint implant, are composed of silicone elastomers with properties of hyperelastic materials [1], which exhibit nonlinear elastic deformation [15]. They exhibit a very large elastic deformation with very little to no plastic deformation before failure. Many materials, including rubber, silicone elastomers, and foam, also exhibit this behavior. Because of the complex mechanical behavior of hyperelastic materials, various models have been formulated to study their dynamics. [16]

Finite element (FE) analysis simulates physical phenomena using numerical mathematical techniques by transforming continuous variables or functions into a discrete form [17]. FE analysis is a key analytical tool in mechanical engineering as well as other fields. As the scope of applications for FE analysis in clinical practice has become diversified along with the progression of FE analysis simulation software, several achievements using FE analysis in biomechanical studies have been realized. The distribution of stress, strain, and deformation can be simulated, and the analysis of solid objects as well as nonlinear materials may be conducted by FE analysis. In the field of orthopedic surgery, FE analysis is frequently used to design orthopedic implants and to evaluate their biomechanical properties. [18]

Several FE analysis studies comparing the Swanson^®^ finger joint implant and the NeuFlex^®^ silicone implant have been published [19–21], however, they studied implants in the metacarpophalangeal joint, not the PIPJ, and were published over 12 years ago. As a result, may have lower numerical analysis speed and higher computational cost of FE analysis compared with current studies using latest engineering simulation programs [22]. Furthermore, studies comparing FH implants and the novel RCJ implant have not been conducted. Therefore, in this study, we compared the biomechanical characteristics during PIPJ range of motion exercise between the conventional PIPJ FH implant and the novel PIPJ RCJ implant using FE analysis.

## Materials and methods

### Configuration and parameters of conventional FH and novel PIPJ implants

**Fig. 1:**
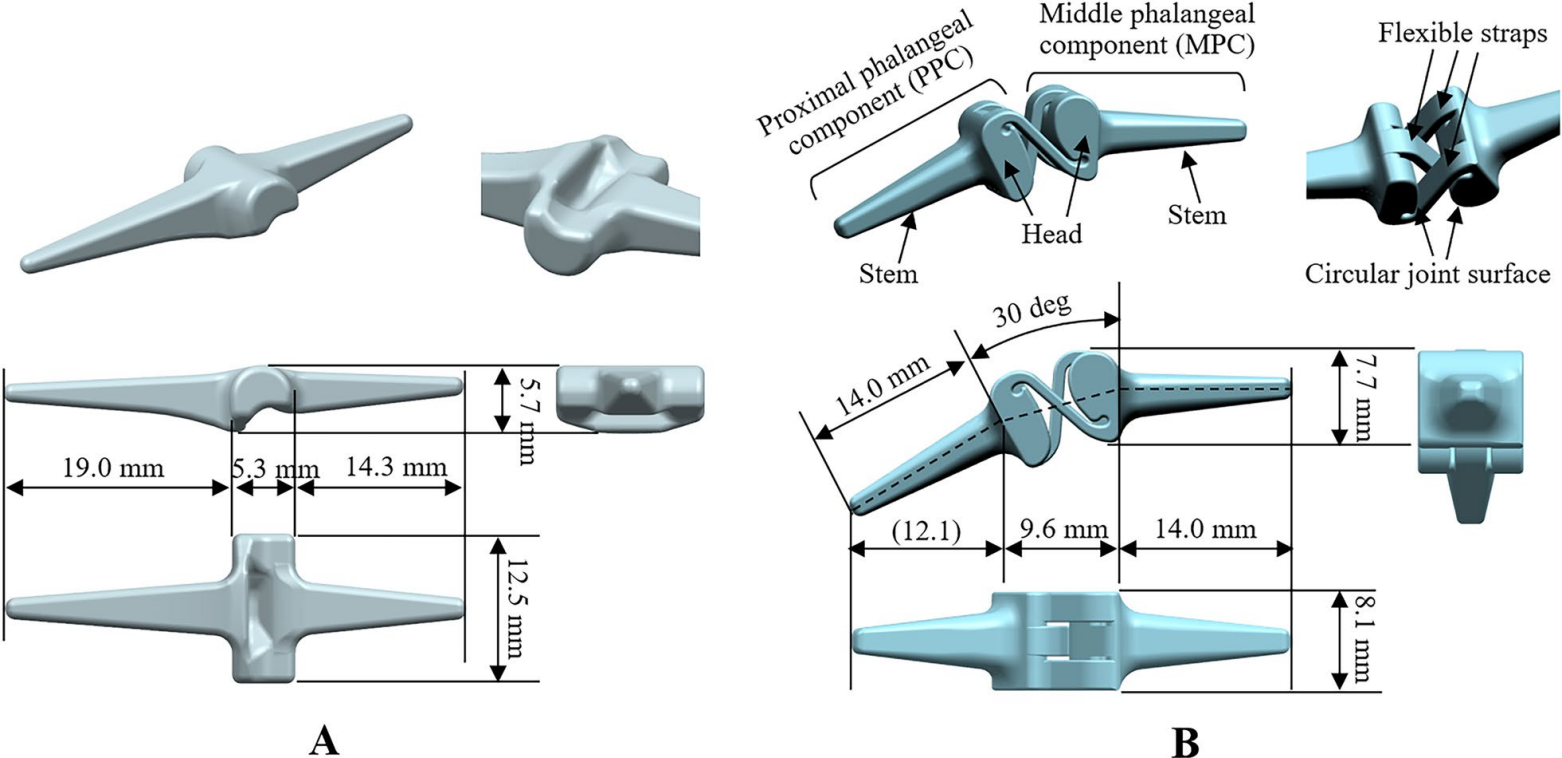
**A**) The dimensions and configurations of the Swanson^®^ silicone finger joint implant. **B**) The dimensions and configurations of the novel PIPJ implant using RCJ mechanism

A Swanson^®^ silicone finger joint implant (Flexspan^®^ size No. 2) was used to evaluate the biomechanical properties of a conventional PIPJ FH implant [23]. A 3D laser surface scanning system [HandySCAN 3D™|SILVER, Creaform Inc., Lévis, QC, (accuracy: 0.03 mm)] was used to obtain the surface shape of the conventional PIPJ FH implant. Figure1a, b shows the dimensions and configurations of the Swanson^®^ silicone finger joint and novel PIPJ implants, respectively. The configuration and parameters of the novel PIPJ implant were adapted from a previous study [12]. This design allows motion around 1 degree of freedom (1-DOF), consisting of PIPJ flexion or extension in the sagittal plane, and includes two components: the middle phalangeal component and proximal phalangeal component. Each component has one stem and one head with a circular joint surface. The components are linked by three flexible straps with equal widths (Fig. 1b). Of these, two straps are symmetrically located in relation to the third strap, which is located at the center. The width of each strap is 2.5 mm, and the thickness is 1 mm. The width of the implant head is 8.1 mm. The PIPJ in the previous study [12] was manufactured using rigid materials for the head and stem as well as flexible woven materials for the straps. The proposed novel PIPJ implant in the present study was fabricated by one silicone material using a molding process, which reduces production cost and provides more flexibility (Additional file 1). To conduct an FE analysis under equal conditions of mechanical dimensions between the two implants, the width, length, and fillet of the two stems of each implant were set similarly. Furthermore, to disregard the interaction between the implant and bone, such as micromotion, and to focus on the simulation of the hinge and straps during flexion and extension motions, we defined the analysis range only up to the junction between the stem and the implant head and excluded the portion of the stem inserted into the medullary canal [24].

This study was an experimental study and did not include human participants or human derived materials. Thus, approval of an institutional review board was not required.

### Determination of material characteristics of conventional FH and novel RCJ implants

The two implants were assumed to have the same material characteristics. Each implant was composed of a silicone elastomer, such as Flexspan^®^, which was assumed to exhibit nearly incompressible hyperelastic behavior. Based on the results of previous study [19], the Arruda–Boyce material model, which closely represents the material properties of conventional PIPJ FH implant, was used. Therefore, the shear modulus μ, limiting network stretch λ, and Poisson’s ratio ν were established at 1 MPa, 4.66, and 0.45, respectively. The thermal expansion coefficient was set to 2.63 × 10^6^ (K^−1^, at 300 K) [25]. The detailed mechanical characteristics are listed in Table 1.

**Table 1:** Material properties of the silicone elastomer used in the present study

Properties	Values
Shear modulus (μ, MPa)	1
Limiting network stretch (λ)	4.66
Poisson’s ratio	0.45
Bulk modulus (MPa)	9.667
Thermal expansion coefficient (K^−1^)*	2.63 × 10^6^

*The thermal expansion coefficient represents the value at 300 K

### Finite element modeling process of each implant

Configuration data of the conventional PIPJ FH and novel PIPJ implants were imported into the NX computer-aided design program (version 8.5, Siemens, Munich, Germany) to generate 3D geometrical surface and hyperelastic models of both implants. The hyperelastic models of both implants were imported into structural engineering software (Ansys Mechanical 2022 R1; Ansys, Inc., Canonsburg, PA) to produce the FE mesh and to perform FE analysis.

**Fig. 2:**
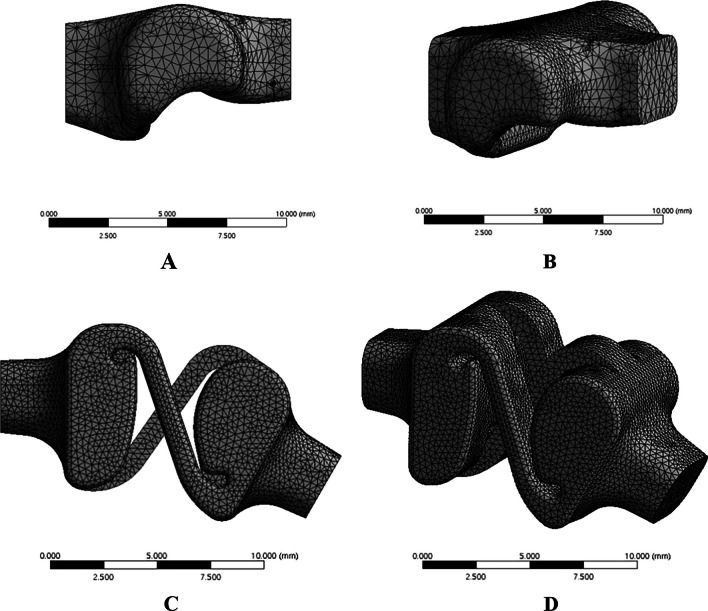
**A**, **B**) The side and perspective views of the mesh for the conventional PIPJ FC implant. **C**, **D**) The side and perspective views for the mesh of the novel PIPJ implant using the RCJ mechanism

The FE mesh models of the conventional PIPJ FH and novel PIPJ implants have 34,474 and 240,962 nodes, respectively. 3D tetrahedron element was used for FE modeling and the average element sizes were set as 0.5 mm and 0.3 mm for the PIPJ FH and novel PIPJ implants, respectively. The mesh sensitivity analyses verified that the FE analysis models in this study have converged to a solution (Additional file 2). The FE mesh models for the heads, hinge and straps without stems were produced as shown in Fig. 2.

### Boundary and loading conditions

The FE analysis modeled the changes of mechanics during the flexion–extension motion of two PIPJ implants. We determined the flexion–extension motion of the PIPJ within the joint range of motion between 0° and 90° incrementally, with reference to the joint range of motion of a normal PIPJ [26]. Because of the difference in the basic design of both implants, the conventional PIPJ FH implant exhibits a 0° flexion position, whereas the novel PIPJ implant exhibits a 30° flexion position when unloaded.

For design comparison, the proximal phalangeal stems of both implants were constrained to zero displacement using the multi–point constraint with rigid behavior. The rotational motion to flexion and extension direction was imposed to the middle phalangeal stems of both implants (Table 2). Rotation in the other directions was constrained; however, the 3-dimensional translational motions were not. Under these conditions, FE solutions were calculated for the von-Mises stress and strain as well as the moment reaction of both implants.

**Table 2:** Loading steps for each implant

	Conventional PIPJ FH implant	Novel PIPJ implant using a RCJ mechanism
Loading step 1	Clockwise rotation of 90° to obtain 90° of flexion (full flexion state)	Counterclockwise rotation of 30° to obtain full extension
Loading step 2	Counterclockwise rotation of 90° to obtain full extension (unloaded state)	Clockwise rotation of 30° to obtain 30° of flexion (unloaded state)
Loading step 3		Clockwise rotation of 60° to obtain 90° of flexion
Loading step 4		Counterclockwise rotation of 60° to obtain 30° of flexion (unloaded state)

Based on the above boundary and loading conditions, the mean values within a total modeled volume and the maximum value of the von-Mises stress and strain of the two implants were analyzed with respect to the PIPJ range of motion from 0° to 90°. The total moment reactions, the sum of the moment reactions of the x, y, and z axes, were also calculated within the same PIPJ range of motions. In addition, within the PIPJ’s functional range of motion between 27° to 86°, [26] the mean values of the mean and maximum values of von-Mises stress and strain as well as the mean value of total moment reactions were also compared. The novel PIPJ implant has a 30° prebending, causing a change in the sign of the moment reaction around the 30°. Therefore, the mean value of the total moment reaction was calculated as the sum of the absolute values of the total moment reaction within the PIPJ’s functional range of motion.

## Results

### von-Mises stress of the two implants

**Fig. 3:**
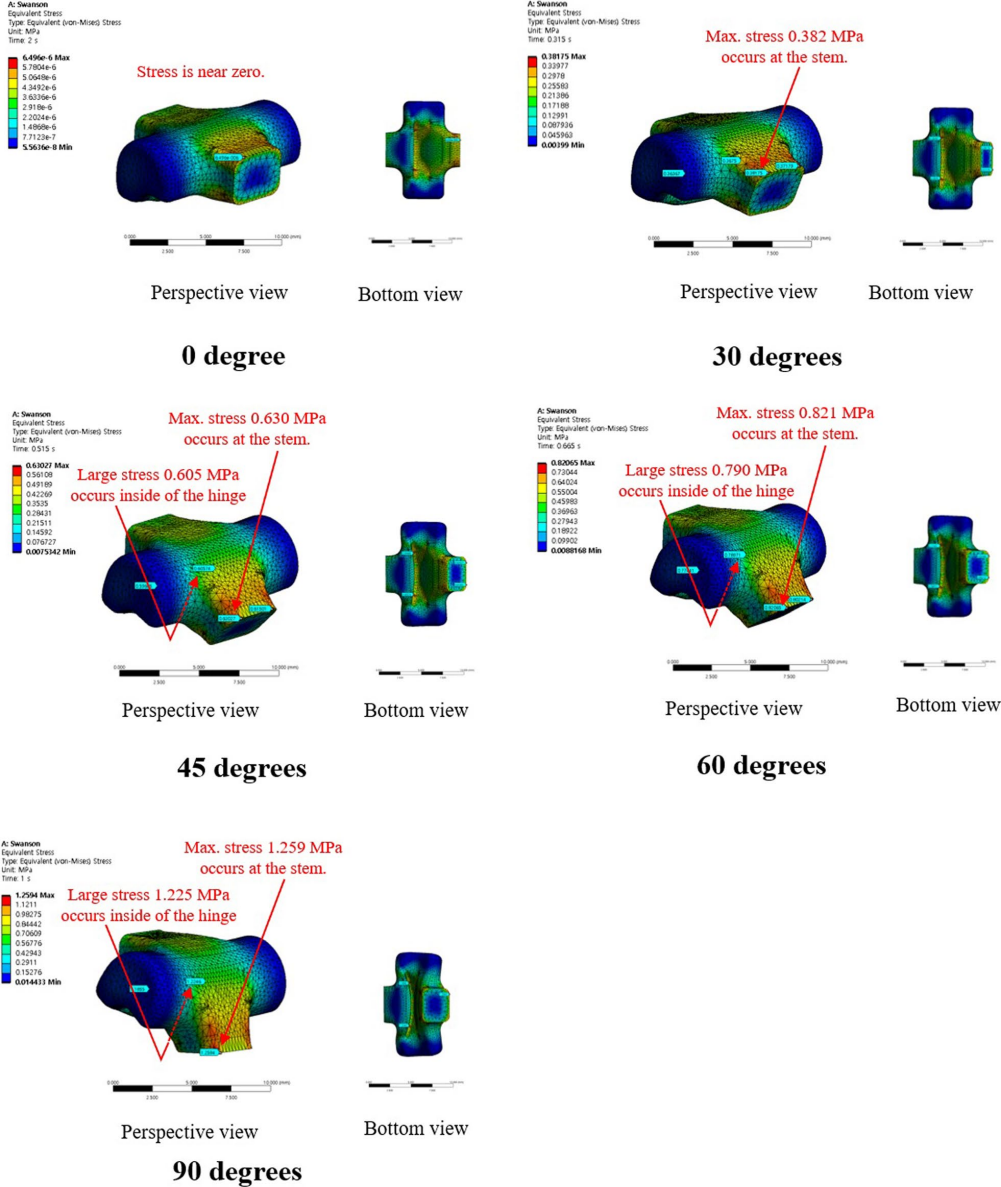
The distribution of von-Mises stress for the conventional PIPJ FH implant at 0°, 30°, 45°, 60°, and 90° of PIPJ flexion

**Fig. 4:**
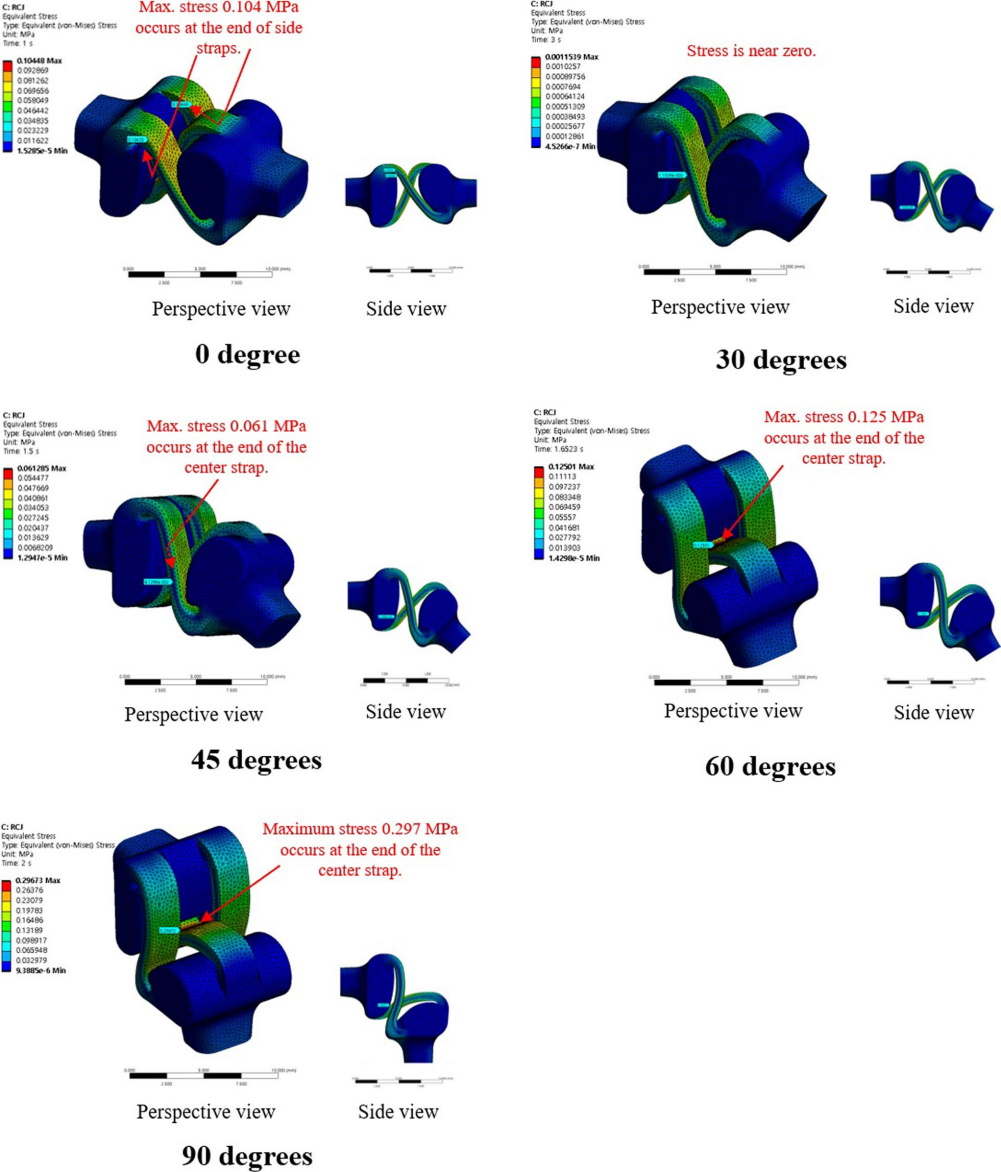
The distribution of von-Mises stress for the novel PIPJ implant at 0°, 30°, 45°, 60°, and 90° of PIPJ flexion

**Fig. 5:**
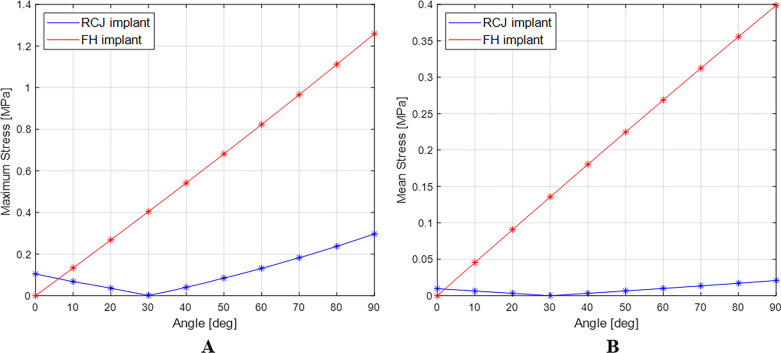
**A**) Line plot depicting the variation of maximum von-Mises stress for the two implants based on the PIPJ flexion angle. **B**) Line plot depicting the variation of mean von-Mises stress for the two implants based on the PIPJ flexion angle

The maximum and mean values of von-Mises stress were 1.26 MPa and 3.99 × 10^−1^ MPa, respectively, at 90° for the conventional PIPJ FH implant and 2.97 × 10^−1^ MPa and 2.09 × 10^−2^ MPa at 90° for the novel PIPJ implant (Figs. 3, 4, 5, Additional file 3). The mean values within the PIPJ’s functional range of motion (27°–86°) of the maximum and mean values for von-Mises stress of the conventional PIPJ FH implant were 7.76 × 10^−1^ MPa and 2.53 × 10^−1^ MPa, respectively, and those of the novel PIPJ implant were 1.21 × 10^−1^ MPa and 0.90 × 10^−2^ MPa. The mean value within the PIPJ’s functional range of motion for the maximum and mean values for von-Mises stress of the novel PIPJ implant was approximately 6.43- and 28.1-fold lower, respectively, compared with that of the conventional PIPJ FH implant (Table 3).

**Table 3:** The mean values within the PIPJ’s functional range of motion (27° to 86°) for the mean and maximum values of the von-Mises stress and strain as well as the mean value of the total moment reactions

	von-Mises stress (MPa)		von-Mises strain (mNm)		Moment reactions (mNm) (Total moment reaction)
	Maximum value	Mean value	Maximum value	Mean value	
Conventional PIPJ FH implant	8.29 × 10^−2^	7.76 × 10^−2^	2.53 × 10^−1^	2.49 × 10^−1^	5.4887
Novel PIPJ implant using a RCJ mechanism	1.21 × 10^−2^	0.30 × 10^−2^	3.85 × 10^−2^	0.90 × 10^−2^	0.1106

*FH* flexible hinge, *PIPJ* proximal interphalangeal joint, *RCJ* rolling contact joint

### von-Mises strain of the two implants

**Fig. 6:**
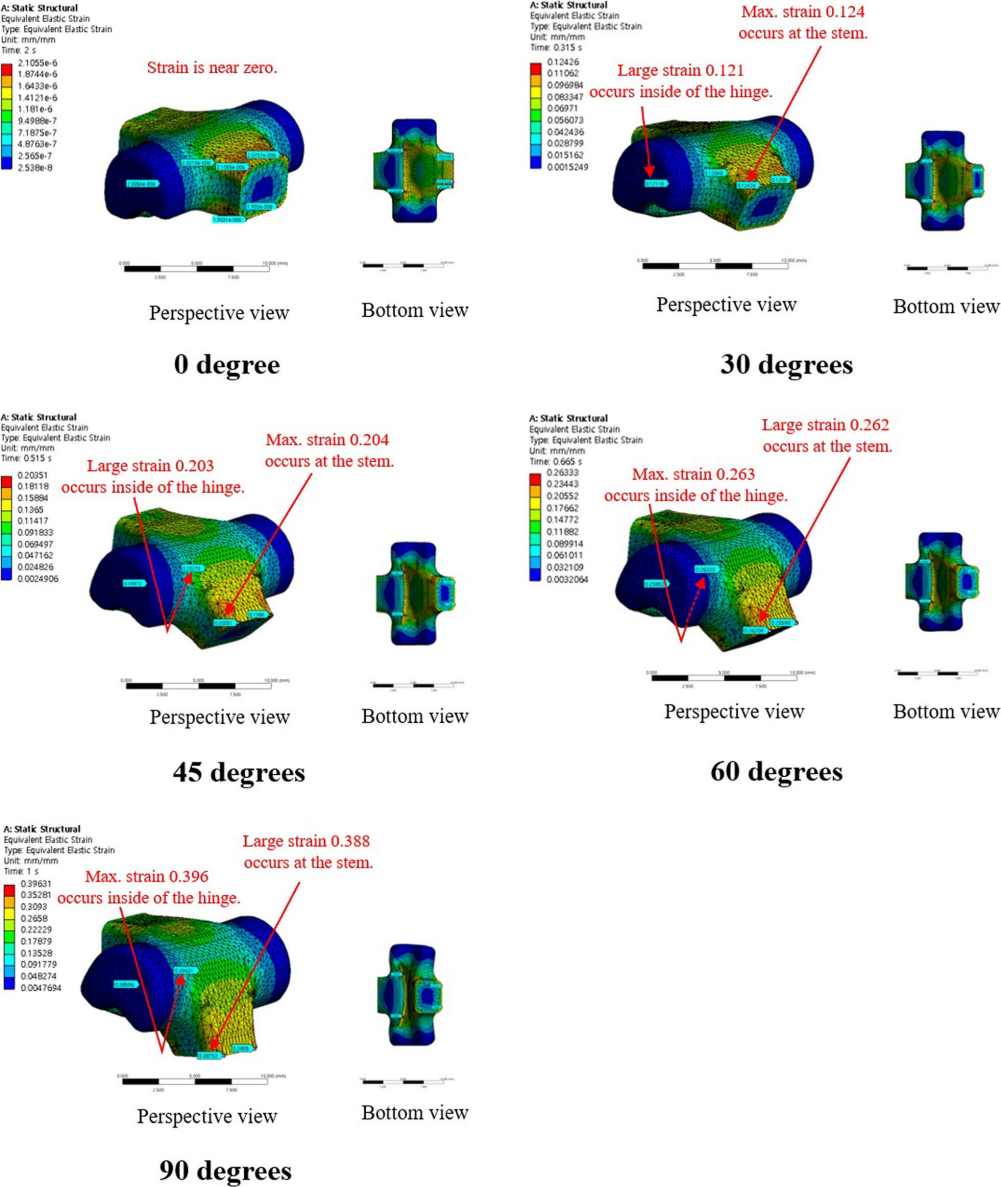
The distribution of von-Mises strain for the conventional PIPJ FH implant at 0°, 30°, 45°, 60°, and 90° of PIPJ flexion

**Fig. 7:**
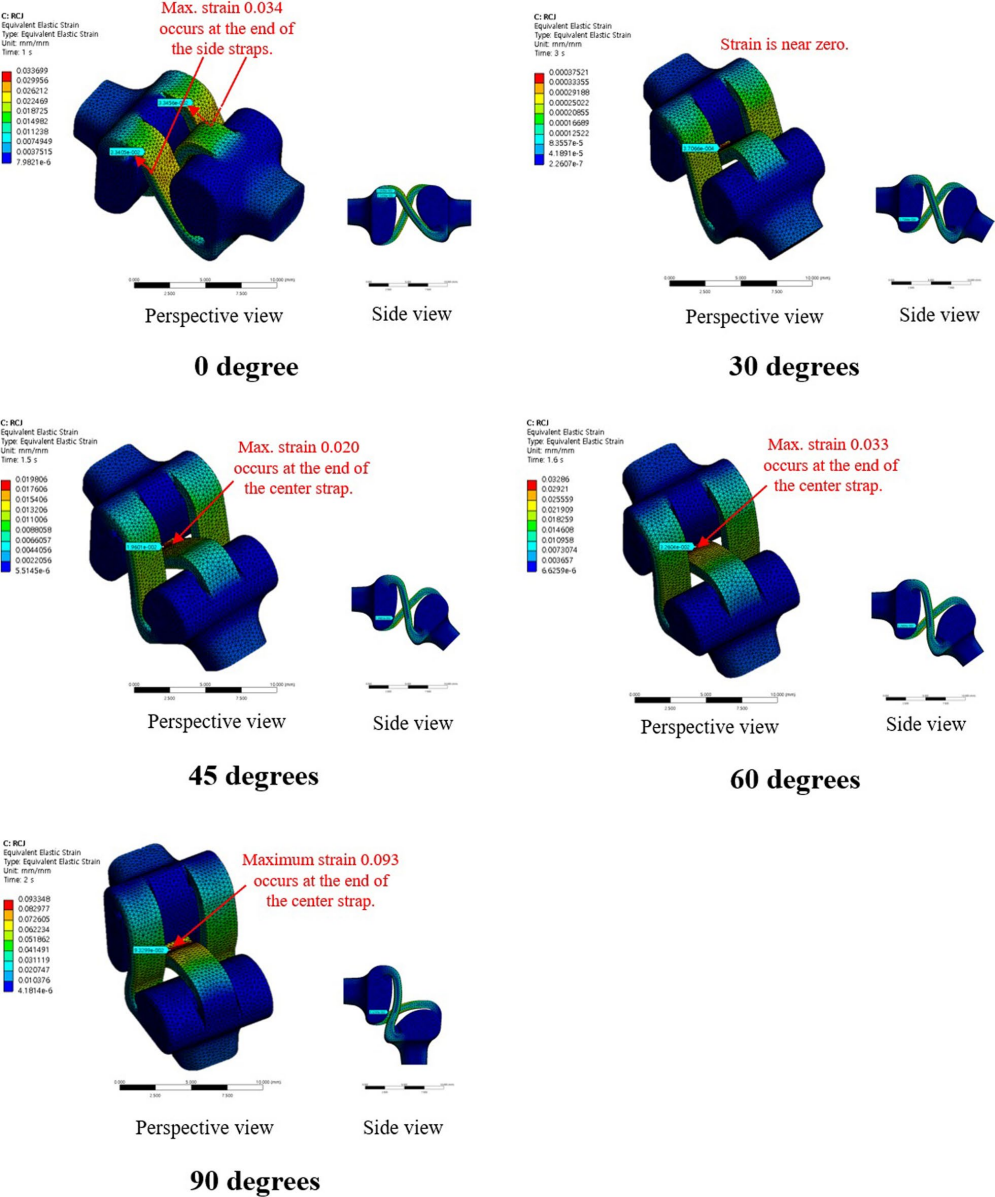
The distribution of von-Mises strain for the novel PIPJ implant at 0°, 30°, 45°, 60°, and 90° of PIPJ flexion

**Fig. 8:**
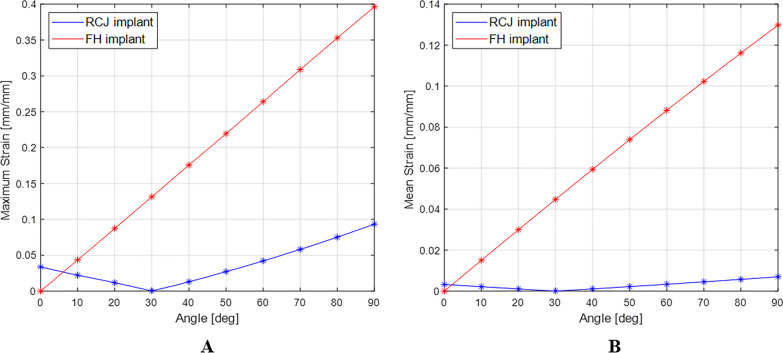
**A**) Line plot depicting the variation of maximum von-Mises strain for the two implants based on the PIPJ flexion angle. **B**) Line plot depicting the variation of the mean von-Mises strain of the two implants based on the PIPJ flexion angle

The maximum and mean values of von-Mises strain were 3.96 × 10^−1^ and 1.30 × 10^−1^ at 90°, respectively, for the conventional PIPJ FH implant, and 9.33 × 10^−2^ and 0.70 × 10^−2^ at 90° for the novel PIPJ implant (Figs. 6, 7, 8, Additional file 4). The mean values within the PIPJ’s functional range of motion for the maximum and mean values of von-Mises strain for the conventional PIPJ FH implant were 2.49 × 10^−1^ and 8.29 × 10^−2^, respectively, and those of the novel PIPJ implant were 3.85 × 10^−2^ and 0.30 × 10^−2^. The mean value within the PIPJ’s functional range of motion for the maximum and mean values of von-Mises strain for the novel PIPJ implant was approximately 6.47- and 27.6-fold lower, respectively, compared with that of the conventional PIPJ FH implant (Table 3).

### Moment reaction of the two implants

**Fig. 9:**
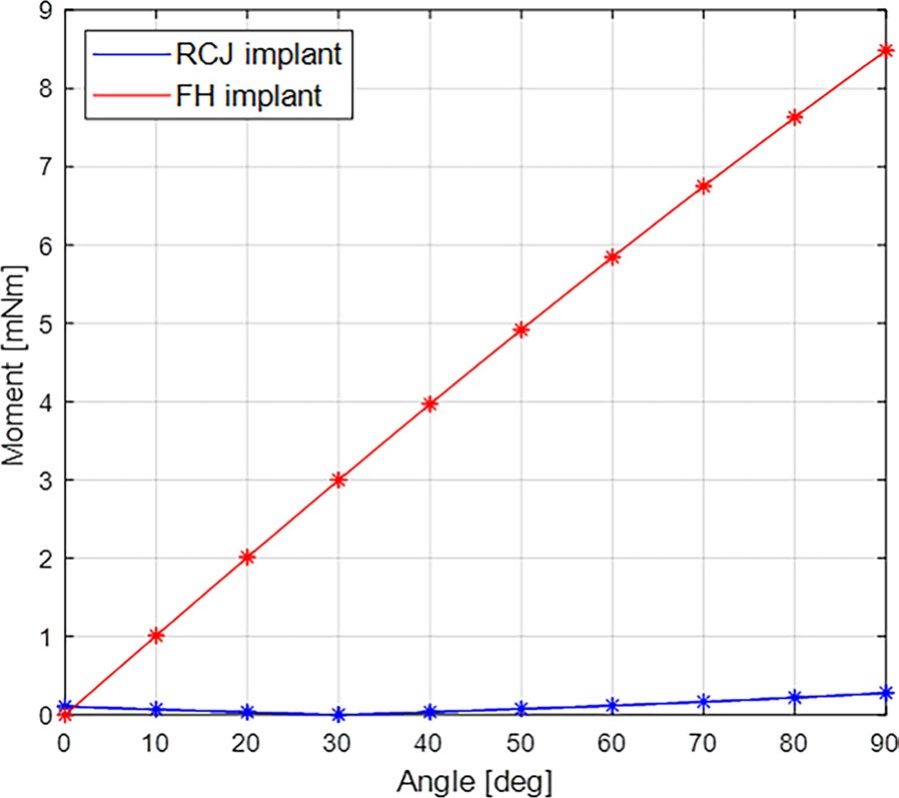
Line plot depicting the variation of the total moment reaction for the two implants based on the PIPJ flexion angle

The maximum value of the total moment reaction of the conventional PIPJ FH implant was 8.484 mNm at 90° compared with that of the novel PIPJ implant was 0.2793 mNm at 90° (Fig. 9**,** Additional file 5). The mean values within a PIPJ’s functional range of motion of the total moment reaction for the conventional PIPJ FH implant and the novel PIPJ implant were 5.4887 mNm and 0.1106 mNm, respectively. The mean value within a PIPJ’s functional range of motion of the total moment reaction for the conventional PIPJ FH implant was approximately 49.6-fold higher compared with that of the novel PIPJ implant (Table 3).

## Discussion

PIPJ FH implants have been widely used as the primary option for PIPJ replacement arthroplasty for several decades; [27] however, they are unable to completely restore the complex and delicate functions of the finger [28]. In addition, metal implants are unconstrained surface replacement implants that offer several advantages, such as a better range of motion. However, because metal implants have critical disadvantages, such as implant loosening and dislocation, they are not widely used as a PIPJ FH implant [29]. Therefore, there is need for a new flexible constrained type of implant that can overcome the limitations of conventional PIPJ FH and unconstrained metal implants.

The proposed novel PIPJ implant allows flexion motions with small stress and moments because of the RCJ formed by the three straps and two circular joint surfaces of the heads. In the RCJ motion, the flexion occurs primarily at the straps and deformation of the stems and heads is substantially small. These straps with a small thickness and wide width can be readily flexed without high stress and simultaneously provide enough stability against loosening and dislocation. The results of the FE analysis for von-Mises stress indicated that the stress distribution for the conventional PIPJ FH implant was concentrated on the hinge region and the cross-sectional plane of the stem during the range of motion. Whereas the novel PIPJ implant using the RCJ mechanism exhibited a relatively even distribution of stress along the strap, with stress concentration observed at the point where the central strap attached to the implant hand. The mean value within PIPJ’s functional range of motion of the mean and maximum value for von-Mises stress of the novel PIPJ implant was approximately 27.6- and 6.43-fold lower compared with that of the conventional PIPJ FH implant. Furthermore, the von-Mises strain distribution of the two implants based on the PIPJ's range of motion, exhibited nearly similar results as the von-Mises stress distribution. These results indicate that cumulative stress generated during repetitive range of motion exercises may be dispersed through the strap, thereby potentially reducing the possibility of fatigue fractures occurring at the link area. Thus, hinge fractures, a critical complication of conventional PIPJ FH implant [1, 27], can be reduced using the novel PIPJ as an alternative. This suggests that the novel PIPJ implant may potentially yield better results in terms of implant longevity compared with the conventional PIPJ FH implant.

Among the similarities between the stress and strain distribution, one difference was observed at the position of the stress and strain points in the FE analysis result for the PIPJ FH implant. While the maximum stress point was at the cross-sectional plane of the stem, the maximum strain point was at the inside of the hinge. It is because the cross-sectional plane was constrained not to deform by the boundary condition of FE analysis. Thus, the plane exhibited large stress without maximum strain. This plane matched the position where the stem is inserted in the medullary canal, a similar constraint restricting deformation occurs, and implant failure occurs [30–32]. However, the precise contact model between the stem and the medullary canal was excluded in the present FE analysis. Therefore, further analysis is warranted.

The results of FE analysis for the moment reaction indicated that the mean value within a PIPJ’s functional range of motion of the total moment reaction for the novel PIPJ implant was approximately 49.6-fold lower compared with that of the conventional PIPJ FH implant. The maximum value of the total moment reaction for the novel PIPJ implant at 90° was 30.38-fold lower compared with that of the conventional PIPJ FH implant. For conventional PIPJ FH implants to achieve small maximum and mean reaction moments similar to those of novel PIPJ implants, the height of the cross-sectional area of the hinge should be approximately 3.7- or 3.1-fold smaller, because the bending moment shows a cube relationship with the height of the cross-sectional area. However, it is inadequate because the small cross-sectional area decreases strength against the large compressive force necessary for grasping or pinching objects. In contrast, the novel PIPJ implant can withstand a large compressive force because of the large cross-sectional area formed by compressed straps between two heads. These results indicate that the elastic rebound strain of the novel PIPJ implant had considerably lower values compared the conventional PIPJ FH implant. Therefore, the novel PIPJ implant inserted with PIPJ can be moved with less driving force compared with the PIPJ with the conventional PIPJ FH implant. Moreover, it also suggests an advantage at achieving a larger range of motion of the PIPJ. Therefore, the novel PIPJ implant using RCJ mechanism may be considered as a valuable alternative to address the issue of limited recovery of range of motion after finger joint replacement arthroplasty. In addition, by enabling a smoother and more natural movement of the hand, it may also contribute to improved hand function. However, the analysis of the aforementioned results may have not contributed to determining implants with optimal physiological conditions for optimal performance.

Several medical-grade silicone elastomers have been used to evaluate small, flexible joint implants [33, 34]. Silicone elastomer is a rubber-like material that exhibits hyperelastic behavior, characterized by large nonlinear reversible elastic deformation [15, 16]. Various hyperelastic material models have been proposed to mimic the characteristics of hyperelastic materials [16]. In this study, the Arruda-Boyce hyperelastic material model was selected to solve the FE model. It is a micromechanical model that describes the deformation behavior of polymeric materials [35]. The Arruda-Boyce model has slower model calculation speed compared with the Neo-Hookian model, but effectively describes strains over 300% [19, 36, 37]. Therefore, it is considered a suitable model for this study, which analyzed silicone elastomers and exhibited large strains during the range of motion process.

This study had several limitations. First, fatigue-type stress, which evaluates the effect of repetitive motions in the daily life on implants, and critical loading could not be simulated in this study. Second, the material properties used in this study are different from those of the real PIPJ FH implant. Therefore, the results from the present study may differ from laboratory tests performed with PIPJ FH implants. Third, because the mechanical loading applied to the PIPJ may vary depending on race, gender, occupation, and lifestyles, various mechanical loading conditions such as traction-compression or varus–valgus loading should be considered. Fourth, the variation in the FE analysis results because of temperature changes was not considered. The strain energy density function of the Arruda-Boyce hyperelastic material model used in the FE analysis of this study was affected by temperature changes. Finally, since this study focused on a comparative analysis of flexible implants with hyperelastic material properties, a comparison between the novel RCJ implant and implants made of different materials, such as pyrocarbon or surface replacement implants, was not conducted.

## Conclusions

To best of our knowledge, this is the first study to compare the biomechanical characteristics of the conventional PIPJ FH implant with a novel PIPJ implant using the RCJ mechanism. The novel PIPJ implant using the RCJ mechanism exhibited a better force distribution and lower moment reaction during range of motion compared with the conventional PIPJ FH implant and may exhibit acceptable longevity. Future studies on the FE analysis, including various loading conditions, and the experimental validation of the FE analysis may help accelerate the clinical application of RCJ mechanism implants in total PIPJ arthroplasty.

### Supplementary Information

**Additional file 1:** **A)** The fabricated novel Rolling Contact Joint (RCJ) implant and four components of molding. **B)** The actual model of RCJ implant made via molding process and its motion during flexion–extension. **C) **The actual model of RCJ implant made via 3D printing and its motion during flexion–extension.

**Additional file 2:** Results of mesh sensitivity test under 15° flexion of proximal interphalangeal joint. **A)** Von-Mises stress of conventional FH implant. **B)** Von-Mises strain of conventional FH implant. **C) **Von-Mises stress of novel RCJ implant. **D)** Von-Mises strain of novel RCJ implant.

**Additional file 3: **The mean values and maximum values of the von-Mises stress for the two implants based on the degrees of PIPJ range of motion

**Additional file 4:** The mean values and maximum values of the von-Mises strain for the two implants based on the degrees of PIPJ range of motion

**Additional file 5:**The total moment reactions for the two implants based on the degrees of PIPJ range of motion

## Data Availability

The datasets used and analyzed during the current study area available from the corresponding author on reasonable request.

## Abbreviations

PIPJ: Proximal interphalangeal joint
FH: Flexible hinge
RCJ: Rolling contact joint
FE: Finite element
3D: Three-dimensional
