# Supplementary material for: Biomechanical comparative finite element analysis between a conventional proximal interphalangeal joint flexible hinge implant and a novel implant design using a rolling contact joint mechanism
Source: J Orthop Surg Res. 2023 Dec 19;18:976. doi: 10.1186/s13018-023-04477-y (PMC10731759; doi:10.1186/s13018-023-04477-y)
Supplement: Supplementary file 1 — Additional file 1: A) The fabricated novel Rolling Contact Joint (RCJ) implant and four components of molding. B) The actual model of RCJ implant made via molding process and its motion during flexion–extension. C) The actual model of RCJ implant made via 3D printing and its motion during flexion–extension. [file 13018_2023_4477_MOESM1_ESM.pptx]

## Slide 1
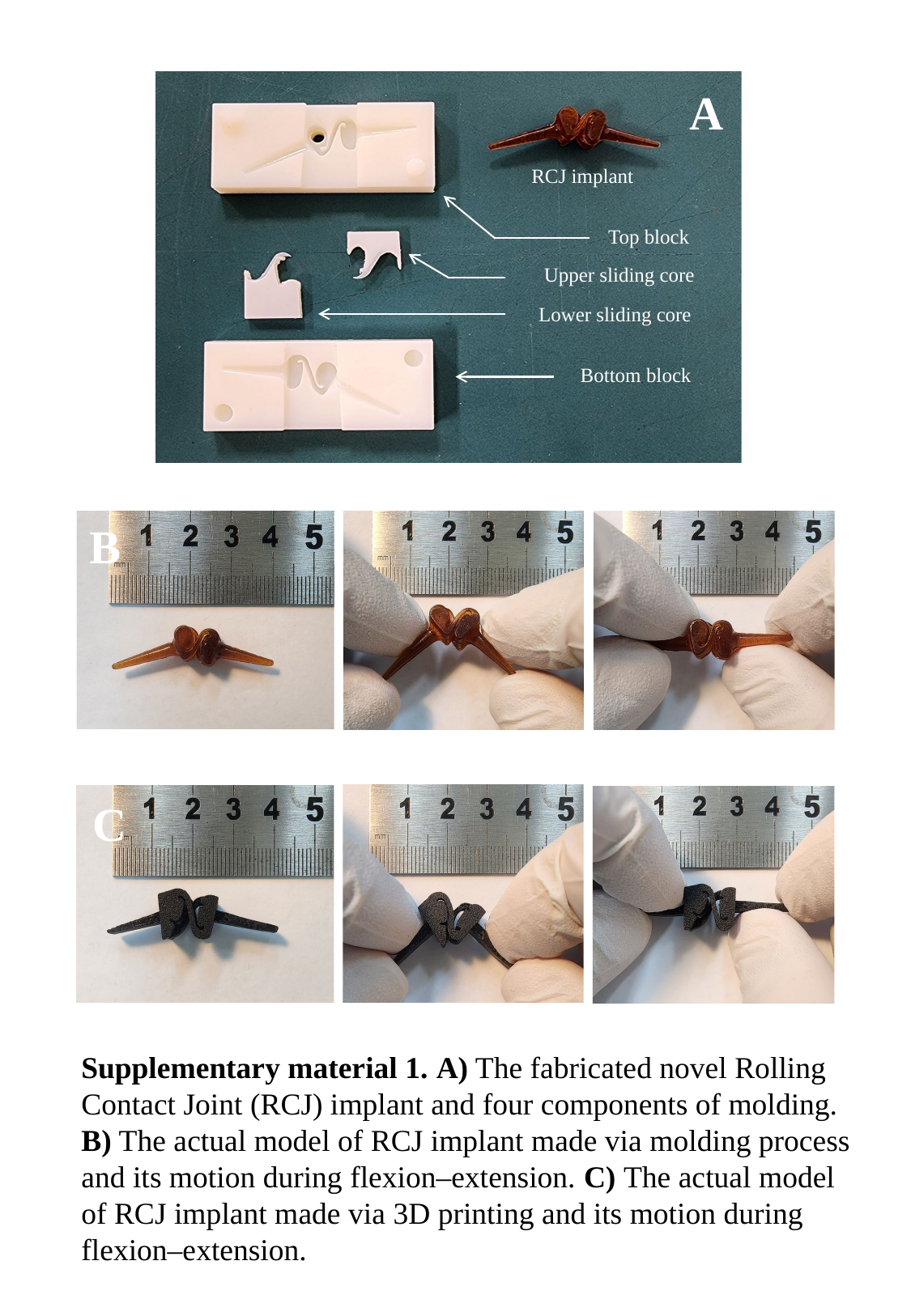

RCJ implant
Top block
Upper sliding core
Lower sliding core
Bottom block
A
B
C
Supplementary material 1. A) The fabricated novel Rolling Contact Joint (RCJ) implant and four components of molding. B) The actual model of RCJ implant made via molding process and its motion during flexion–extension. C) The actual model of RCJ implant made via 3D printing and its motion during flexion–extension.
