# Supplementary material for: Biomechanical comparative finite element analysis between a conventional proximal interphalangeal joint flexible hinge implant and a novel implant design using a rolling contact joint mechanism
Source: J Orthop Surg Res. 2023 Dec 19;18:976. doi: 10.1186/s13018-023-04477-y (PMC10731759; doi:10.1186/s13018-023-04477-y)
Supplement: Supplementary file 2 — Additional file 2: Results of mesh sensitivity test under 15° flexion of proximal interphalangeal joint. A) Von-Mises stress of conventional FH implant. B) Von-Mises strain of conventional FH implant. C) Von-Mises stress of novel RCJ implant. D) Von-Mises strain of novel RCJ implant. [file 13018_2023_4477_MOESM2_ESM.pptx]

## Slide 1
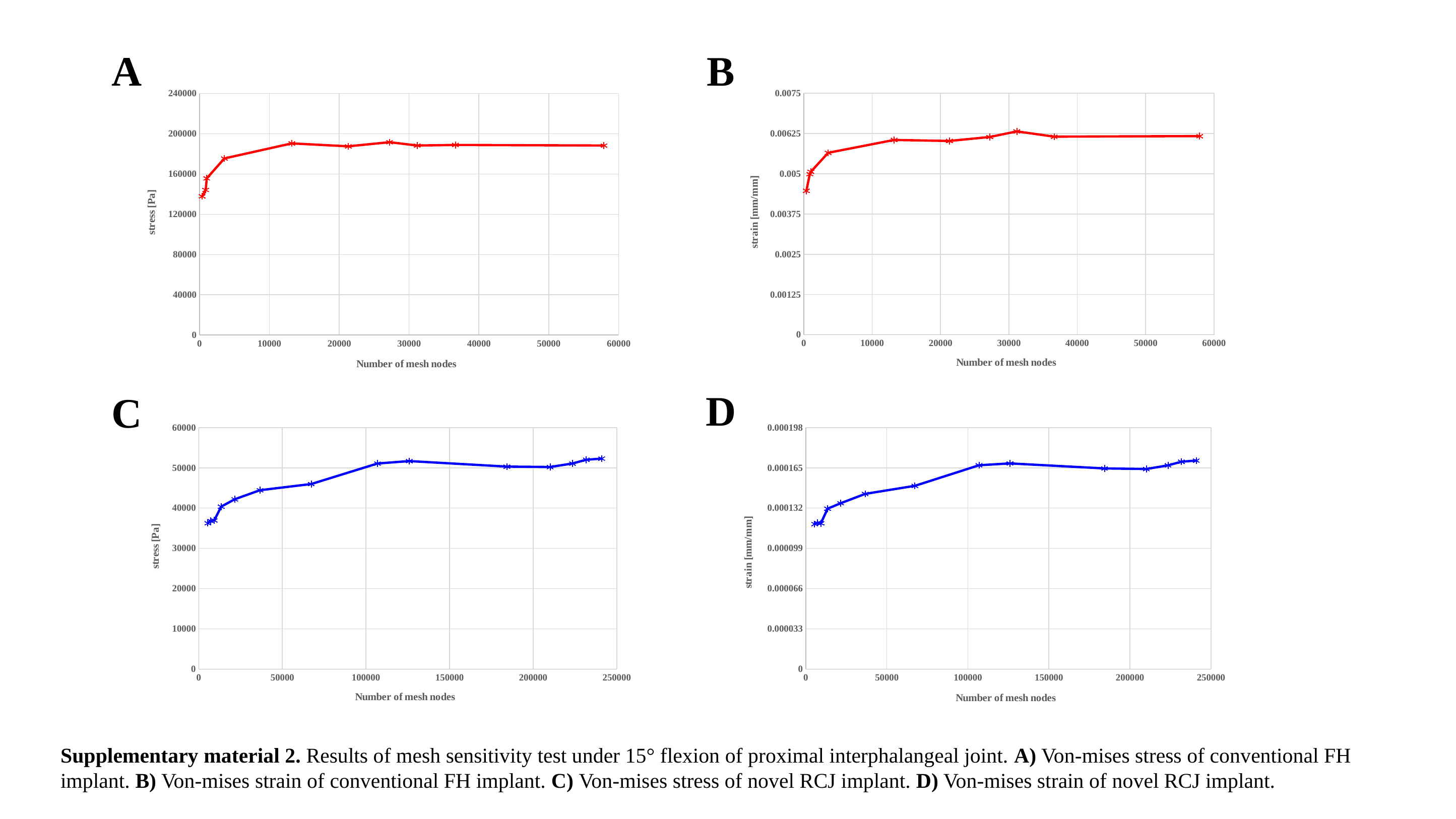

### Chart
| Category | FH implant |
|---|---|A
### Chart
| Category | stress [Pa] |
|---|---|B
A
B
### Chart
| Category | |
|---|---|
### Chart
| Category | RCJ implant |
|---|---|D
C
C
Supplementary material 2. Results of mesh sensitivity test under 15° flexion of proximal interphalangeal joint. A) Von-mises stress of conventional FH implant. B) Von-mises strain of conventional FH implant. C) Von-mises stress of novel RCJ implant. D) Von-mises strain of novel RCJ implant.
