# Supplementary material for: Biomechanical comparative finite element analysis between a conventional proximal interphalangeal joint flexible hinge implant and a novel implant design using a rolling contact joint mechanism
Source: J Orthop Surg Res. 2023 Dec 19;18:976. doi: 10.1186/s13018-023-04477-y (PMC10731759; doi:10.1186/s13018-023-04477-y)
Supplement: Supplementary file 3 — Additional file 3: The mean values and maximum values of the von-Mises stress for the two implants based on the degrees of PIPJ range of motion [file 13018_2023_4477_MOESM3_ESM.docx]

**Supplementary material 3.** The mean values and maximum values of the von-Mises stress for the two implants based on the degrees of PIPJ range of motion.

| **PIPJ Flexion angle** | **Conventional PIPJ FH implant** | | **Novel PIPJ implant using a RCJ mechanism** | | |
| --- | --- | --- | --- | --- | --- |
|  | **Maximum value** | **Mean value** | | **Maximum value** | **Mean value** |
| 0° | 0 | 0 | | 1.04 × 10^−1^ | 0.97 × 10^−2^ |
| 10° | 1.34 × 10^−1^ | 4.55 × 10^−2^ | | 6.77 × 10^−2^ | 0.64 × 10^−2^ |
| 20° | 2.68 × 10^−1^ | 9.08 × 10^−2^ | | 3.57 × 10^−2^ | 0.32 × 10^−2^ |
| 30° | 4.04 × 10^−1^ | 1.36 × 10^−1^ | | 0 | 0 |
| 40° | 5.42 × 10^−1^ | 1.80 × 10^−1^ | | 4.03 × 10^−2^ | 0.32 × 10^−2^ |
| 50° | 6.81 × 10^−1^ | 2.25 × 10^−1^ | | 8.45 × 10^−2^ | 0.66 × 10^−2^ |
| 60° | 8.23 × 10^−1^ | 2.69 × 10^−1^ | | 1.31 × 10^−1^ | 0.99 × 10^−2^ |
| 70° | 9.66 × 10^−1^ | 3.12 × 10^−1^ | | 1.82 × 10^−1^ | 1.35 × 10^−2^ |
| 80° | 1.11 | 3.56 × 10^−1^ | | 2.37 × 10^−1^ | 1.71 × 10^−2^ |
| 90° | 1.26 | 3.99 × 10^−1^ | | 2.97 × 10^−1^ | 2.09 × 10^−2^ |

The tabulated values have units of MPa, FH: flexible hinge, PIPJ: proximal interphalangeal joint, RCJ: rolling contact joint
