# Supplementary material for: Biomechanical comparative finite element analysis between a conventional proximal interphalangeal joint flexible hinge implant and a novel implant design using a rolling contact joint mechanism
Source: J Orthop Surg Res. 2023 Dec 19;18:976. doi: 10.1186/s13018-023-04477-y (PMC10731759; doi:10.1186/s13018-023-04477-y)
Supplement: Supplementary file 4 — Additional file 4: The mean values and maximum values of the von-Mises strain for the two implants based on the degrees of PIPJ range of motion [file 13018_2023_4477_MOESM4_ESM.docx]

**Supplementary material 4.** The mean values and maximum values of the von-Mises strain for the two implants based on the degrees of PIPJ range of motion.

| **PIPJ Flexion angle** | **Conventional PIPJ FH implant** | | **Novel PIPJ implant using a RCJ mechanism** | | |
| --- | --- | --- | --- | --- | --- |
|  | **Maximum value** | **Mean value** | | **Maximum value** | **Mean value** |
| 0° | 0 | 0 | | 3.37 × 10^−2^ | 0.32 × 10^−2^ |
| 10° | 4.36 × 10^−2^ | 1.50 × 10^−2^ | | 2.22 × 10^−2^ | 0.22 × 10^−2^ |
| 20° | 8.74 × 10^−2^ | 3.00 × 10^−2^ | | 1.16 × 10^−2^ | 0.11 × 10^−2^ |
| 30° | 1.31 × 10^−1^ | 4.48 × 10^−2^ | | 0 | 0 |
| 40° | 1.76 × 10^−1^ | 5.94 × 10^−2^ | | 1.30 × 10^−2^ | 0.11 × 10^−2^ |
| 50° | 2.19 × 10^−1^ | 7.39 × 10^−2^ | | 2.72 × 10^−2^ | 0.22 × 10^−2^ |
| 60° | 2.64 × 10^−1^ | 8.82 × 10^−2^ | | 4.21 × 10^−2^ | 0.33 × 10^−2^ |
| 70° | 3.09 × 10^−1^ | 1.02 × 10^−1^ | | 5.82 × 10^−2^ | 0.45 × 10^−2^ |
| 80° | 3.53 × 10^−1^ | 1.16 × 10^−1^ | | 7.52 × 10^−2^ | 0.57 × 10^−2^ |
| 90° | 3.96 × 10^−1^ | 1.30 × 10^−1^ | | 9.33 × 10^−2^ | 0.70 × 10^−2^ |

The tabulated values have units of mNm, FH: flexible hinge, PIPJ: proximal interphalangeal joint, RCJ: rolling contact joint
