# Supplementary material for: Biomechanical comparative finite element analysis between a conventional proximal interphalangeal joint flexible hinge implant and a novel implant design using a rolling contact joint mechanism
Source: J Orthop Surg Res. 2023 Dec 19;18:976. doi: 10.1186/s13018-023-04477-y (PMC10731759; doi:10.1186/s13018-023-04477-y)
Supplement: Supplementary file 5 — Additional file 5:The total moment reactions for the two implants based on the degrees of PIPJ range of motion [file 13018_2023_4477_MOESM5_ESM.docx]

**Supplementary material 5.** The total moment reactions for the two implants based on the degrees of PIPJ range of motion.

| **Flexion angle** | **Conventional PIPJ FH implant** | **Novel PIPJ implant using a RCJ mechanism** |
| --- | --- | --- |
| 0° | 0 | − 0.1067 |
| 10° | 1.0124 | − 0.0699 |
| 20° | 2.0128 | − 0.0348 |
| 30° | 2.9985 | 0 |
| 40° | 3.9671 | 0.0364 |
| 50° | 4.9165 | 0.0759 |
| 60° | 5.8450 | 0.1186 |
| 70° | 6.7496 | 0.1668 |
| 80° | 7.6303 | 0.2202 |
| 90° | 8.484 | 0.2793 |

The tabulated values have units of mNm, FH: flexible hinge, PIPJ: proximal interphalangeal joint, RCJ: rolling contact joint.
